# Use of a self-rating scale of the nature and severity of symptoms in Parkinson’s Disease (PRO-PD): Correlation with quality of life and existing scales of disease severity

**DOI:** 10.1038/s41531-017-0021-5

**Published:** 2017-06-16

**Authors:** Laurie K. Mischley, Richard C. Lau, Noel S. Weiss

**Affiliations:** 1 Bastyr University Research Institute, Kenmore, WA USA; 2 School of Biological and Population Health Sciences, Oregon State University, Corvalis, OR USA; 3 Department of Epidemiology, University of Washington School of Public Health, Seattle, WA USA

## Abstract

A self-rating scale was developed to permit patient-reported, remote assessment of Parkinson’s disease symptom severity. The goal was to create a continuous outcome measure that does not require a clinical exam, does not fluctuate in response to dopaminergic medications, takes only a few minutes to complete, allows for stratification by symptom(s), and captures both motor and non-motor Parkinson’s disease symptoms, major contributors to quality of life. The Patient Reported Outcomes in Parkinson’s Disease (PRO-PD) is the cumulative score of 32 slider bars, each evaluating a common Parkinson’s disease symptom. The PRO-PD has been used as an outcome measure in three studies. The baseline data from each of these studies were pooled for this analysis. Symptom frequency and severity are described, as well as correlation coefficients with existing measures of Parkinson's disease severity. Data on 1031 participants with Parkinson's disease were available for analysis. Fatigue, impaired handwriting, daytime sleepiness, slowness, tremor, muscle cramps, and forgetfulness were the most frequently reported symptoms. Persons with a relatively long duration of Parkinson's disease tended to report more, and more severe, symptoms. The PRO-PD was most highly correlated with the Parkinson’s Disease Questionaire-39 (*r*  =  0.763, *P*  <  0.000) and Patient-Reported Outcome Measurement Information System Global quality of life (*r * =  −0.7293, *P*  <  0.000), other patient-reported quality of life measures. The PRO-PD_non-motor_ subset was highly correlated with the Non-Motor Symptom Score (*r*  =  0.7533, *P*  <  0.000). There was a moderate correlation seen with Hoehn & Yahr (*r*  =  0.5922, *P*  <  0.000), total Unified Parkinson’s disease Rating Scale (*r*  =  0.4724, *P*  <  0.000), and the Timed-Up-&-Go (*r*  =  0.4709, *P*  <  0.000). The PRO-PD may have utility for patients, providers, and researchers as a patient-centered measure of Parkinson’s disease symptom severity. Further PRO-PD validation efforts are warranted.

## Symptom assessment tools: Tracking disease progression

Scientists develop a novel Patient Reported Outcomes in Parkinson’s Disease (PRO-PD) scale to improve the assessment of disease progression. To overcome the limitations of existing measures of Parkinson's disease (PD) severity, Laurie K Mischley at Bastyr University Research Institute, USA, and colleagues have created a simple and low cost self-reporting scale that captures subtle changes in both motor and non-motor symptoms. A key feature is that it asks patients to estimate the severity of 32 common symptoms, on average, over the previous 7 days. This reduces score fluctuations due to patients’ response to medication and thus allows better detection of the underlying disease progression. PRO-PD scores from 1031 participants with PD increased with illness duration and correlated well with established scales of disease severity and other patient-reported quality of life measures.

## Introduction

While advances have been made in the management of PD motor symptoms, little progress has been made in identifying therapies capable of reducing the rate of disease progression.^1^ The current lack of disease modification therapies may be due, in part, to limitations in biomarkers and outcome measures that aptly assess symptom status and progression. Two main measures of PD progression exist: the Hoehn and Yahr scale and the Unified Parkinson’s Disease Rating Scale (UPDRS). The Hoehn & Yahr (HY) scale scores ranging only from 1 to 5 and it can take years before the disease progresses enough to change score.^2^ Additionally, concerns have been raised about the Hoehn and Yahr’s ability to adequately capture non-motor as well as more subtle variations in disease progression.^3^ The HY scale is commonly supplanted by the UPDRS, which for decades has been the most widely used outcome measure for PD symptom severity in clinical trials. However, the UPDRS was developed when PD was thought to be a predominantly motor disease, and incapacitating symptoms such as constipation, fatigue, and impaired sleep are inadequately captured. While several of these limitations were addressed in the updated MDS-UPDRS,^4^ both UPDRS and MDS-UPDRS scores are responsive to dopaminergic medications.^5, 6^ While responsiveness to symptomatic therapies is a advantageous attribute for a scale to have if the goal is to evaluate the impact of that treatment, this quality is disadvantageous if the goal is to detect disease progression over time, since adequate symptom management may conceal the underlying disease progression.

**Fig. 1:**
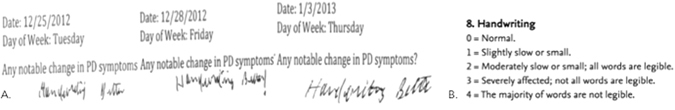
Subjective assessment. An example of participant-reported improvement in handwriting and the subjective nature of dysgraphia assessment using Unified Parkinson’s Disease Rating Scale (UPDRS) as an outcome measure. **a**. Journal entry from the Phase I/II a study of intrnanasaal glutathione in PD. **b**. UPDRS: Question about handwriting from UPDRS

Motor symptoms occur relatively late in the course of PD, with non-motor symptoms often occurring over a decade prior to motor onset.^7^ To effectively target upstream interventions, an outcome measure is needed that captures early and non-motor symptoms (e.g., constipation, anosmia, handwriting), allows for descriptions of symptom diversity, and is directly correlated with meaningful change among those affected. Figure 1 exemplifies how difficult it is to translate participant experience to UPDRS scores, where robust changes are required to detect differences.

PRO measures have historically been applied to provide the patient’s own assessment of their symptoms, quality of life, and functional status.^8^ PROs have been shown to be underutilized and inconsistently included endpoints in clinical trials that assess symptoms.^9, 10^ To better understand the patient’s perpective related to treatment success, expectations, and importance of motor and non-motor domains, the Patient-Centered Outcomes Questionnaire was administered to 181 individuals with PD. The authors conclude the definition of treatment success should be broadened to include the patient’s perspective.^11^ The Neuro-QOL, an outcome measure developed by the NINDS to assess the self-reported measures on health-related quality of life of adults with neurological disorders, was designed to overcome many of these limitations.^12^ While the Neuro-QOL is a valuable addition to PD clinical trials, it is not specific enough for PD to permit stratification by symptoms.

Although several PRO measures for PD symptoms exist, all outcome measures have inherent strengths and limitations. The Parkinson’s Disease Questionnaire (PDQ-39) has been shown to correlated with the Medical Outcome Study 36-Item Short Form Health Survey (SF-36), a generic measure of quality of life, although the PDQ-39 scale is most useful in a more severe disease.^13^ The Patient-Reported Outcome tool for Advanced Parkinson’s Disease (PRO-APD)^14^ was developed to better understand patient perceptions and expectations of treatment. Designed to document symptoms late in the disease and providing realistic counseling to patients, it offers little ability to identify symptoms that are present early in the disease. As a paper assessment tool with Likert scales numbered 0 to 7, the PRO-APD requires scoring by researchers and is less responsive to change than a slider scale with a continuum from 1 to 100. The SCOPA-Motor Scale can be completed in approximately half the time of the UPDRS, and has been shown to be a consistent and valid measure of motor function, disability, and complications of therapy.^15^ The SCOPA-Motor requires a clinical exam by a trained provider and does not address common and debilitating non-motor symptoms, limiting its utility as a single outcome measure for the comprehensive assessment of PD. The Self-Assessment PD Disability Scale assesses disability in nine daily activities and is targeted toward patients living at home.^16^ The PD Activities of Daily Living Scale assesses difficulty of accomplishing daily activities due to PD; while it generates a single global rating, it cannot be stratified by symptom(s). While useful for evaluating individual symptoms, The Parkinson Fatigue Scale-16, the PD Sleep Scale, and the Sialorrhea Clinical Scale for PD, and the Freezing of Gait Questionnaire do not capture global disease severity. The non-motor questionnaire (NMSQuest) was the first self-completed non-motor questionnaire for PD. The NMSQuest is a screening tool for non-motor symptoms, it is not a rating instrument and does not provide an overall disability score. As a non-motor scale, it neglects motor symptoms.^17, 18^ The PDQ-39 is the most extensively used and tested instrument for quality of life in PD; like the others, it is not continuous, stratifiable by symptom, and inadequately captures motor and non-motor symptom diversity. An ideal outcome measure would incorporate a spectrum of wellness, as well as disease; of the available measures, only the Patient-Reported Outcome Measurement Information System (PROMIS) lacks this ceiling effect.

We attempted to develop a PD-specific PRO that overcame some of the limitations of the HY, UPDRS, MDS-UPDRS, and Neuro-QOL. The PRO-PD was designed to be an inexpensive and easily administered assessment tool capable of quantifying symptom incidence and severity in a clinically relevant fashion.

We sought to evaluate whether PRO-PD scores increase with increasing duration of PD, whether or not higher scores correlate with quality of life measures, and the degree to which the cumulative PRO-PD score correlates with existing measures of PD severity.

## Results

Demographic and other characteristics of the 1031 participants are listed in Table 1. The average duration of PD, as measured by years since diagnosis, was 5.0  +  5.2 years. Participants had a mean PRO-PD score of 637 at diagnosis, and those with longer durations of illness had relatively higher scores—an average increase of 33 points per year (95% CI: 28, 39).

**Table 1:** Demographic and selected other characteristics of study participants

Participant Characteristics	
Pooled Datasets	*N* = 1031
CAM Care in PD	971 (94%)
Phase IIb (in)GSH	43 (4%)
CNS Uptake of (in)GSH	15 (2%)
Gender	
Male	478 (47%)
Female	544 (53%)
Missing	9
Mean age (standard deviation), years	63.2 (9.4)
Years Since Diagnosis (SD)	5.0 (5.2)
Estimated Hoehn & Yahr	
1: 1-sided symptoms only, minimal disability	486 (51.2%)
2: Both sides affected, balance is stable	154 (16.2%)
3: Mild to moderate disability, balance affected	268 (28.2%)
4: Severe disability, able to walk and stand without help	31 (3.3%)
5: Confinement to bed or wheelchair unless aided	2 (0.2%)
Unknown	9 (0.9%)
Missing	81
Ethnicity	
Caucasian	925 (95.8%)
Hispanic	13 (1.4%)
Asian/ Pacific Islander	9 (0.9%)
Black	6 (0.6%)
Native American	2 (0.2%)
Other	11 (1.1%)
Missing/Unknown	47
Income	
Less than $20,000	52 (5.7%)
Between $20–40,000	139 (15.1%)
Between $40–60,000	135 (14.7%)
Between $60–80,000	139 (15.1%)
Between $80–100,000	128 (13.9%)
Between $100–150,000	181 (19.7%)
More than $150,000	147 (16.0%)
Missing/Unknown	110
Education	
Grades 9–11	13 (1.4%)
Completed High School/GED	100 (10.4%)
Technical school certification	49 (5.1%)
Associate’s Degree	70 (7.3%)
Bachelor’s Degree	277 (28.7%)
Graduate/professional degree	453 (46.9%)
Missing/Unknown	65

Missing data are excluded from percentage calculations.

The frequency and severity of individual symptoms, and their relation to duration of PD, are presented in Table 2. The most frequently reported symptoms were fatigue (92%), impaired handwriting/ typing (91%), daytime sleepiness (89%), fatigue (92%), slowness (88%), muscle pain/ cramping (88%), tremor (88%), and impaired memory (87%). All symptoms, save tremor and nausea, were reported more commonly in persons whose PD had been present for a relatively longer period of time. (Table 2)

**Table 2:** PRO-PD severity by symptom and years since diagnosis

PRO-PD Scores Following PD Diagnosis							
PD Symptom	Number (%) reporting symptom (*N* = 1029)	0–5 years after diagnosis Mean (SD) (*n* = 642)	5–10 years after diagnosis Mean (SD) (*n* = 227)	10–15 years after diagnosis Mean (SD) (*n* = 85)	15 + years after diagnosis Mean (SD) (*n* = 77)	Estimated yearly increase Mean (95% CI)	*P*-value
Fatigue	92%	33.1 (24.3)	40.0 (23.1)	50.5 (25.1)	47.4 (24.6)	1.19 (0.91, 1.48)	<0.000
Handwriting	91%	32.4 (24.2)	44.8 (25.5)	55.3 (27.0)	51.0 (29.3)	1.61 (1.31, 1.91)	<0.000
Daytime sleepiness	89%	27.9 (23.3)	36.0 (22.5)	45.9 (25.8)	43.1 (24.7)	1.18 (0.9, 1.46)	<0.000
Slowness	88%	25.9 (21.8)	34.6 (20.4)	46.6 (21.9)	41.9 (23.1)	1.3 (1.04, 1.56)	<0.000
Tremor	88%	26.4 (20.9)	30.4 (21.5)	28.5 (23.4)	29.4 (26.4)	0.23 (−0.04, 0.49)	0.089
Muscle cramps	88%	27.2 (24.0)	35.4 (25.4)	45.2 (26.8)	35.7 (26.5)	0.98 (0.68, 1.27)	<0.000
Memory/forgetfulness	88%	27.5 (21.0)	32.7 (21.8)	37.4 (20.8)	34.6 (23.2)	0.58 (0.33, 0.84)	<0.000
Sense of balance	86%	21.0 (19.6)	28.8 (19.8)	40.9 (23.2)	43.3 (26.5)	1.51 (1.26, 1.76)	<0.000
Sense of smell	85%	45.2 (32.8)	49.3 (32.0)	55.2 (29.8)	53.2 (32.9)	0.72 (0.33, 1.10)	<0.000
Sexual dysfunction	82%	33.2 (30.3)	40.8 (30.7)	47.8 (29.7)	46.8 (30.8)	1.01 (0.65, 1.38)	<0.000
Urinary functions	82%	25.9 (24.7)	37.4 (26.7)	44.3 (26.3)	45.0 (30.4)	1.52 (1.21, 1.83)	<0.000
Stooped posture	82%	21.8 (18.7)	30.4 (21.3)	36.8 (22.9)	35.8 (20.7)	1.09 (0.85, 1.32)	<0.000
Walking	82%	18.8 (18.0)	27.1 (20.0)	40.3 (21.4)	33.6 (22.4)	1.24 (1.01, 1.47)	<0.000
Anxiety	79%	22.1 (22.9)	24.4 (22.9)	37.5 (27.3)	28.5 (25.4)	0.74 (0.46, 1.02)	<0.000
Insomnia	77%	26.2 (26.1)	31.5 (27.3)	46.5 (28.0)	32.9 (26.4)	1.01 (0.69, 1.33)	<0.000
Motivation and Initiative	77%	23.4 (22.8)	27.1 (23.0)	34.0 (23.5)	32.7 (24.3)	0.61 (0.33, 0.88)	<0.000
Speech	77%	18.2 (18.6)	29.2 (21.9)	40.1 (24.5)	32.5 (23.3)	1.32 (1.08, 1.57)	<0.000
Rising from seated	76%	18.4 (18.9)	25.9 (19.2)	36.5 (21.1)	33.9 (22.8)	1.1 (0.86, 1.33)	<0.000
Dressing, grooming, eating	73%	14.8 (16.7)	23.3 (18.4)	32.8 (21.4)	28.6 (20.5)	1.15 (0.94, 1.36)	<0.000
Constipation	73%	20.3 (21.8)	27.4 (24.2)	31.2 (22.5)	31.6 (25.4)	0.85 (0.58, 1.12)	<0.000
Depression	73%	17.4 (19.4)	20.6 (21.7)	26.0 (22.0)	21.5 (20.7)	0.38 (0.13, 0.62)	0.002
Loss of interest	71%	19.2 (21.6)	22.5 (22.7)	28.5 (23.2)	27.5 (25.1)	0.56 (0.3, 0.83)	<0.000
Comprehension	69%	16.0 (18.6)	21.5 (20.2)	24.9 (21.2)	25.2 (21.4)	0.66 (0.43, 0.89)	<0.000
Sleep behavior disorder	65%	19.3 (24.2)	27.7 (26.5)	34.3 (27.2)	35.8 (26.1)	1.26 (0.96, 1.56)	<0.000
Drooling	65%	16.7 (20.5)	26.7 (23.9)	34.6 (24.8)	31.4 (24.8)	1.17 (0.91, 1.43)	<0.000
Restless legs	64%	18.7 (24.3)	24.7 (25.6)	31.3 (26.1)	27.8 (26.4)	0.79 (0.49, 1.08)	<0.000
Dizzy on standing	64%	14.6 (19.7)	19.5 (20.9)	23.7 (24.7)	24.5 (23.8)	0.6 (0.35, 0.85)	<0.000
Visual disturbance	58%	12.3 (17.9)	21.5 (23.7)	30.8 (26.8)	22.5 (23.2)	1.01 (0.75, 1.26)	<0.000
Falling	55%	11.0 (17.1)	18.0 (21.9)	30.1 (24.0)	29.2 (28.3)	1.35 (1.12, 1.59)	<0.000
Dyskinesia	47%	8.8 (16.7)	22.5 (23.0)	33.2 (27.0)	30.7 (25.7)	1.73 (1.49, 1.97)	<0.000
Freezing	47%	9.4 (16.1)	16.6 (20.5)	26.5 (24.3)	26.9 (26.8)	1.14 (0.92, 1.37)	<0.000
Nausea	43%	9.6 (17.0)	12.6 (18.9)	15.7 (20.2)	10.5 (15.0)	0.19 (−0.03, 0.4)	0.084
Hallucinations	31%	4.6 (10.2)	10.1 (16.1)	15.3 (19.6)	14.2 (21.4)	0.73 (0.56, 0.89)	<0.000

**Fig. 2:**
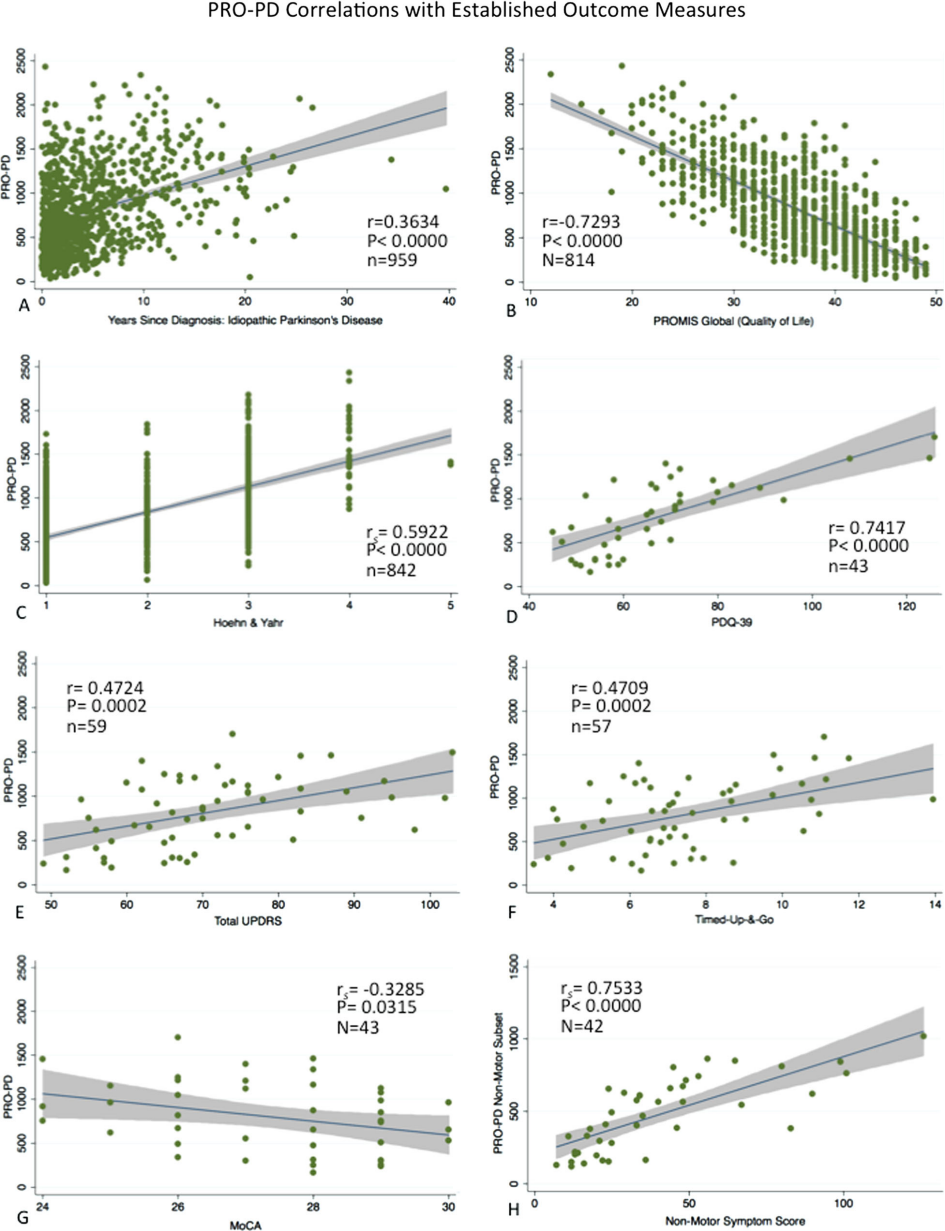
PRO-PD correlation with existing measures of PD status: PRO-PD correlation with **a** years since diagnosis, **b** PROMIS Global Quality of Life, **c** Patient reported Hoehn and Yahr (HY), **d** PDQ-39, **e** Unified Parkinson’s Disease Rating Scale (UPDRS), **f** Montreal Cognitive Assessment, **g** Timed-Up & Go (TUG), and **h** Non-Motor Symptom Scale (NMSS)

The PRO-PD was highly correlated with the PDQ-39 (*r*  = 0.763, *P * <  0.000) and the PROMIS Global QoL (*r*  = −0.7293, *P*  <  0.000). The PRO-PD_non-motor_ subset was also highly correlated with the NMSS (*r*  = 0.7533, *P*  < 0.000). PRO-PD correlated moderately with Hoehn & Yahr (*r* = 0.5922, *P* < 0.000). There was a low correlation between years since diagnosis (*r*  = 0.3634, *P*  <  0.000), total UPDRS (*r*  = 0.4724, *P * <  0.000), TUG (*r*  = 0.4709, *P*   <  0.000), and the MoCA (*r*  = −0.3285, *P*  <  0.000). (Figure 2).

## Discussion

At the National Institute of Neurological Disorders and Stroke Parkinson’s Disease 2014 conference, one of the top ten recommendations to further clinical research of PD was to find innovative high-quality measurements to assess symptoms in PD.^19^ We believe the development of the PRO-PD is one step towards that goal and offers a simple, low cost means to measure both the motor and non-motor symptoms of PD.

There are several limitations to this measurement. The PRO-PD is a cumulative score and all symptoms are weighted equally. Symptom severity does not always translate to debilitation, e.g., moderate scores for constipation, fatigue, or handwriting can be among the most debilitating aspects of PD for some individuals. The current version of the PRO-PD requires computer access and literacy, and thus study participants tended to be better educated than typical PD patients. Hoehn and Yahr scores are patient reported for most subjects, and although previous research has demonstrated a high level of agreement between patient and clinician rated disability scores,^20^ discrepancies may still exist. The small sample size on which some correlations are based is a limitation; correlations observed in small samples should be interpreted cautiously. Further validation efforts are still needed, including assessment of internal consistency, test-retest reliability, content validity (including face validity), construct validity, structural validity, cross-cultural validity, criterion validity, responsiveness, and Interpretability.^21^

The results of this study show that the PRO-PD correlates well with previously established PD measures such as the HY, UPDRS, PDQ-39, PROMIS Global Quality of Life, Non-Motor Symptom Score (NMSS), and Timed-Up & Go (TUG). As the survey asks individuals to estimate symptom severity, on average, over the past week, it is not susceptible to daily fluctuations that other measures are susceptible when performed during the window of a clinic visit. These strengths highlight PRO-PD’s potential value as a simple, low cost means of evaluating PD progression that can be done remotely and does not require the presence of a trained clinician. The data used to generate the PRO-PD can be obtained remotely, an advantage over measures that require the participant to commute to the clinician’s office, often a difficult task for individuals with a movement disorder. Generating a PRO-PD score requires no clinician time.

The PRO-PD non-motor subset score, which correlates well with the NMSS, offers a simple means of evaluating the non-motor symptoms of PD, which are often overlooked and difficult to measure. This is especially critical because of the recognition that the motor symptoms of PD often do not occur until late in the disease’s progression, where critical neurological damage may have already been done.^22^ The data presented in Table 2 indicate that non-motor symptoms are at least as prevalent and severe as motor symptoms, and also suggest they are being poorly treated. These findings are in line with other studies that have also concluded that non-motor symptoms of PD have been reported by patients to be the most debilitating and are closely correlated with the patient’s report of their quality of life.^23, 24^

Unlike other available patient-reported scales, the PRO-PD can be stratified by symptom, which is useful to researchers working to describe subgroups by phenotype, or therapeutic developers attempting to describe the domains for which an intervention is most helpful. Already, it is being used in clinic waiting rooms; providers can use a printed version to rapidly identify the symptoms that need to be addressed during the clinic visit, and it can be scanned into electronic medical records (EMR). Eventually, it may be possible for participants to fill it out from home prior to their visit and auto-populate EMR. Providers and patients can use the score to see how the patient compares to others diagnoses the same number of years ago, set goals for improving symptoms, and identify when there has been an abrupt worsening. The PRO-PD provides a novel currency that can be used by patients, clinicians, and researchers.

## Methods

Three studies have utilized the PRO-PD as an outcome measure and all three datasets were combined for these analyzes. The first was an internet-based natural history study, “CAM Care-PD”, for which the PRO-PD was originally developed. This project was designed to describe modifiable lifestyle variables associated with the slowest rate of progression.^25^ The baseline data from all enrolled participants between the dates of March 2014 and January 2017 (*n*   =  971); individuals enrolled in the ongoing CAM Care-PD and are referred to throughout this manuscript at “Cohort 1”. The size of this dataset was its strength, although these data are limited by the survey design and corresponding inability to perform a physical exam, confirm a diagnosis, a lack of corresponding objective data, and reliance on the individuals’ ability to assign his/her own HY score. Only individuals with idiopathic PD were used in this analysis (*n*  =  971) (Table 1); 181 individuals were exclude who claimed to have “parkinsonism”, multiple system atrophy, progressive supranuclear palsy, Picks disease, or another diagnosis.

To compensate for the limitations in the data quality available from Cohort 1, these data were merged with PRO-PD data obtained from two clinical trials. For both of these studies, the PRO-PD was collected on the same visit that PD symptom status and severity using traditional outcome measures by a trained study clinician. Cohort 2 is a pharmacokinetic study (*N*  = 15), and Cohort 3 is a phase IIb study of intranasal glutathione in PD, a ongoing randomized PD clinical trial (RCT).^26^ Two individuals participated in both studies; only their first set of data was used (*n*  =  43). MoCA scores >25 were a requirement of study inclusion for both of clinical trials, administered to all 60 clinical trial participants by a trained physician during screening procedures. These combined datasets were used to describe the association between PRO-PD and years since diagnosis, patient estimated HY scores, UPDRS, NMSS,^27^ Parkinson’s Disease Questionaire-39 scores (PDQ-39),^28^ MoCA,^29^ and quality of life among participants.

The Bastyr University and/or University of Washington IRB approved all studies and all subjects provided informed consent. Individuals for the CAM Care in PD study were recruited via the internet and community outreach. For trials that collect these data at multiple time points, only baseline data is reported here. All analyzes are cross-sectional.

**Fig. 3:**
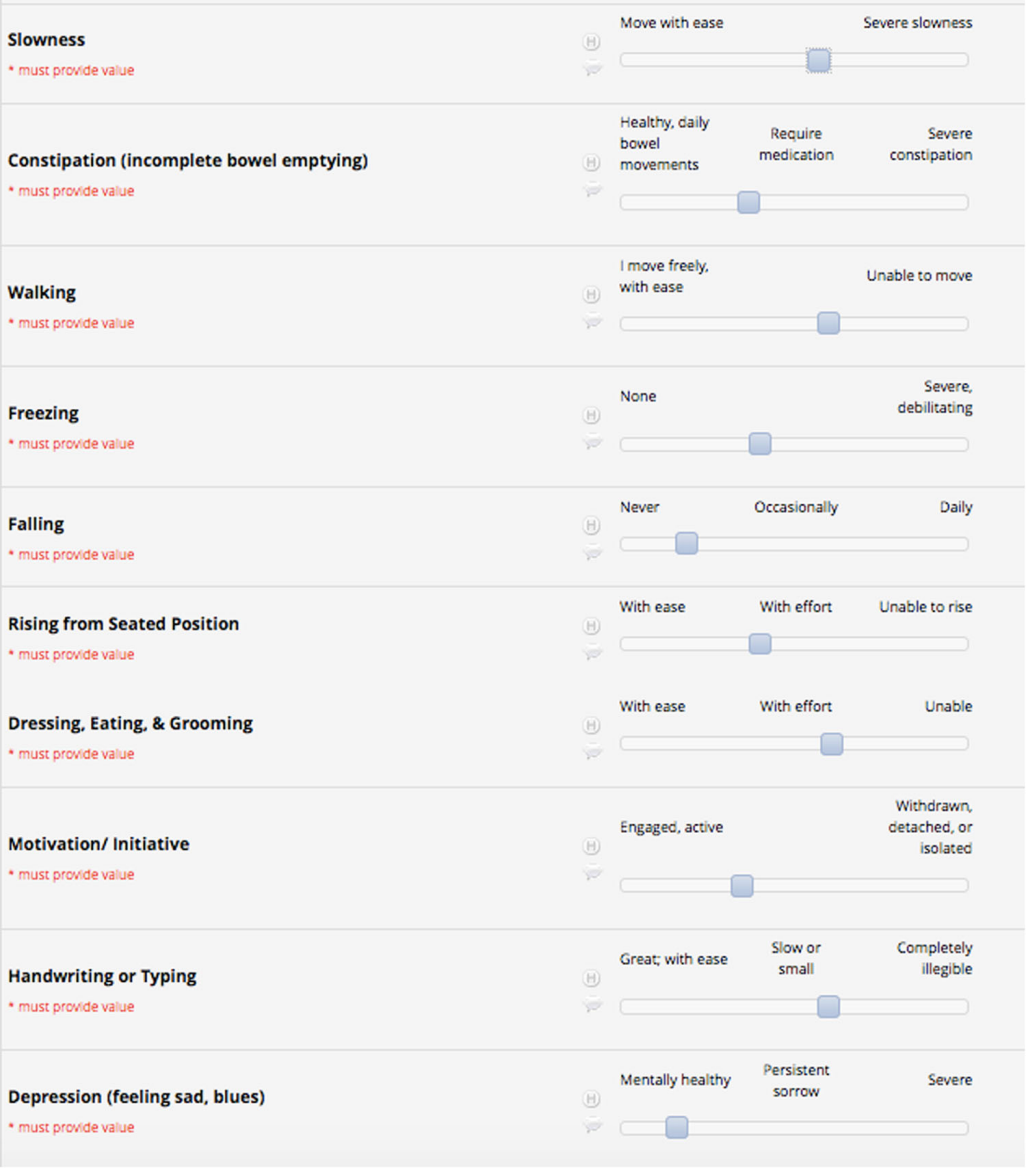
Patient experience: Individuals touch anywhere on the bar to describe the severity of each symptom. The cumulative score of all 32 variables is the PRO-PD score

The PD symptoms chosen for inclusion in the PRO-PD were based on clinical experience, patient feedback, and a literature review of the topic. One of the authors, L.K.M., has more than a decade of clinical experience caring for individuals with PD and developed the list over several months, generating variables for inclusion as patients described symptoms and concerns. The list was modified with exposure to conference presentations, publications related to PD phenotypes, and conversations with colleagues regarding symptom picture in PD. L.K.M. chose the symptoms predicted to have the least amount of overlap (to minimize time/ survey burden) and provide the most diverse snap-shot of disease heterogeneity. For each of the 32 identified symptoms, the participant was provided a slider bar, with the far left side of the bar always representing a lack of symptom or sense of wellness in that domain, and the far right end of the bar representing maximum severity. (Figure 3) The participant was given the instruction to rate each symptom based on their experience, on average, over the 7 days prior. Anchor terms are placed at the both ends and the mid-point of the slider bar, so that all individuals are identically oriented. The participant did not see a numerical score, although their placement of the tab on the bar translates to a score between 0 and 100, with the higher number always reflecting greater symptom severity. The rationale for not showing individuals the number on the slider bar is to make the outcome measure as simple as possible and limit the amount of time it takes to complete it; the more there is to consider, the longer it takes to complete the questionnaire. The sum of these symptoms generated the total PRO-PD score. 19 of these symptoms, identified as non-motor symptoms, were separately classified as a subscore, PRO-PD_non-motor_. PRO-PD_non-motor_ used for comparison with the NMSS, was defined a priori to include the following symptoms: constipation, lack of motivation, depression, loss of interest, anxiety, fatigue, daytime sleepiness  +  temperature dysregulation, orthostatic hypotension, visual disturbances, insomnia, REM sleep behavior disorder, muscle pain, drooling, memory impairment, comprehension disability, hyposmia, sexual dysfunction, urinary dysfunction, and hallucinations (personal communication, K. R. Chaudhuri, 2015).

Study data were collected and managed using Research Electronic Data Capture (REDCap) tools hosted at Bastyr University and the University of Washington.^1^ REDCap is a secure, web-based application designed to support data capture for research studies, providing: 1) an intuitive interface for validated data entry; 2) audit trails for tracking data manipulation and export procedures; 3) automated export procedures for seamless data downloads to common statistical packages; and 4) procedures for importing data from external sources.

To determine symptom frequency, a participant was said to have the symptom if he/ she reported a score for that symptom ≥5. An early version of the survey made answering symptom severity questions optional. For the first 971 responses, there were an average 15 skipped responses for each symptom, ranging from 10 missed (hallucinations) to 40 (memory). Because the PRO-PD is a cumulative score, individuals who skipped one of the variables were assigned the mean score for that variable. To improve data integrity moving forward, IRB approval was obtained to make all symptoms mandatory, essential for the cumulative score.

Linear regression and Pearson correlation coefficients were used to describe associations between PRO-PD and years since diagnosis, UPDRS, PDQ-39, and PROMIS Global quality of life (QoL) measures. The PROMIS Global Health assessment tool was developed by the National Institutes of Health; it was designed to be relevant across a range of conditions, enabling efficient application of patient-reported outcomes across clinical trials and practice.^30, 31^ The Timed-Up-and-Go (TUG) was performed using an iPhone 5 in pocket of the individual and software by Objective Movement Disorder Measurement System 2.0 OMDM Mobility^32^ (Kinetics Version 1.4.1, Connexed Technologies). A chair with arms was placed in a hallway, and a piece of tape 3 meters from the chair was placed on the floor. Participants were asked to remain seated until they were instructed by the software to rise, walk as quickly as possible to the tape, turn around, and return to the chair and sit down. The motion sensor on the phone stopped the clock when the participant resumed a seated position. Linear regression and the Pearson correlation coefficient were used to compare the non-motor subset of the PRO-PD with the NMSS. Logistic regression and the Spearman correlation coefficient were used to describe associations between PRO-PD and HY. Per convention, 0–.1: no correlation, 0.1–0.3: weak correlation, 0.3–0.5: low correlation, 0.5–0.7: moderate correlation, 0.7–0.9: high correlation; 0.9–1.0: very high correlation.^33^ Data analyzes were conducted using Stata 13.1 for Mac (StataCorp, College Station, TX). Analysis of ten self-reported questions related to global health derived from the PROMIS; the summary scores from all ten items comprised the PROMIS Global scores using the recommended scoring method.^31^
